# Evaluation of Protective Coatings for High-Corrosivity Category Atmospheres in Offshore Applications

**DOI:** 10.3390/ma12081325

**Published:** 2019-04-23

**Authors:** Ainara López-Ortega, Raquel Bayón, José Luís Arana

**Affiliations:** 1 IK4-TEKNIKER, Tribology Unit, Iñaki Goenaga 5, 20600 Eibar, Spain; raquel.bayon@tekniker.es; 2 Department of Metallurgical and Materials Engineering, University of the Basque Country, 48013 Bilbao, Spain; jl.arana@ehu.es

**Keywords:** offshore, corrosion, coatings, weathering

## Abstract

The interest in renewable energies obtained from the resources availed in the ocean has increased during the last few years. However, the harsh atmospheric conditions in marine environments is a major drawback in the design of offshore structures. The protective systems that are employed to preserve offshore steel structures are regulated by several standards (ISO 12944, NORSOK M-501), which classify the corrosivity category of offshore installations as C5-M and Im2. In this work, three coatings employed in offshore components protection have been evaluated according to these standards by performing weathering aging tests in different climatic cabinets. The coatings studied were a thermally sprayed carbide coating with an organic sealant (C1), a thermally sprayed aluminum (TSA) coating with an organic topcoat (C2), and an epoxydic organic coating reinforced with ceramic platelets (C3). The only coating that reached the higher categories in all the tests was the C2 coating. The C1 coating presented ferric corrosion products coming from the substrate in some of the tests, and blistering was detected in the C3 coating.

## 1. Introduction

**Figure 1:**
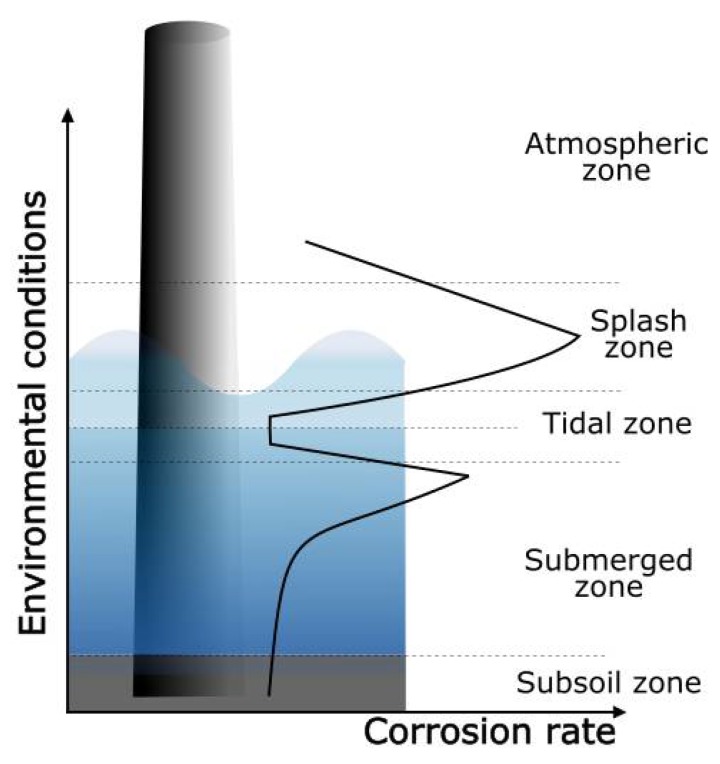
Zones of corrosion and relative corrosion rate depending on the exposure zone.

The marine environment is a very aggressive working atmosphere, where structural materials and components are exposed to ultraviolet radiation, a chloride-rich salty environment, frequent wetting and drying cycles, high humidity, the attack of biological microorganisms and marine bacteria, etc. [1,2,3,4,5]. Furthermore, there is also abrasion and severe wear caused by sand, ocean currents, floating wastes, and contamination [1,5,6,7,8]. As schematically depicted in Figure 1, offshore materials and structures are exposed to five corrosion zones, with different material corrosion rates [3,6,9,10,11]:

- **Atmospheric zone:** This zone is located above the sea level, and the severity of corrosion is related to the time of wetness, during which electrochemical processes take place. There is a direct relationship between atmospheric salt content and corrosion rate. Materials are also exposed to solar radiation, which deteriorates the performance of organic coatings.
- **Splash zone:** This section in the structure is intermittently wetted, due to tides and the wind. The corrosion rate of metals in this zone is the highest, due to the aerated condition, which makes the access of dissolved oxygen for electrochemical reactions easy. Since it is continuously being wetted, chlorides can concentrate on the surface while the water films dry. Furthermore, the impinging of seawater containing sand and other flowing matter adds a mechanical component to the materials’ deterioration in this exposure zone.
- **Tidal zone:** The materials are alternately submerged and exposed to the splash zone, as the tide fluctuates. In the submerged condition, materials are exposed to a well-aerated seawater, which favors the attachment and growth of biofouling. The corrosion rate is influenced by the tidal flow, with higher corrosion rates with increasing movements.
- **Submerged zone:** The section of the structure that is always immersed in the sea. The corrosion rate in this zone depends on the availability of oxygen to be transported to the cathodic sites of the materials’ surfaces. As oxygen concentration varies with depth, decreasing with increasing distance to the surface, the corrosion rate is also slower at higher depths.
- **Subsoil:** In the buried structure, the oxygen concentration is low, and hydrogen sulfide may be present.

The most employed structural material in offshore applications is steel—usually mild or low-alloyed—due to its superior mechanical properties [1,12,13]. However, this kind of steels possess low corrosion resistance in seawater and corrode relatively fast. The corrosion rate of mild steel in seawater has been measured to be 250 µm per year [6,14]. In order to avoid or prevent premature failures due to the high corrosion rate of structural steel, coatings have successfully been used. The requirements of protective coatings and their application procedures vary significantly depending on the installation location, i.e., onshore or offshore. Currently, the protection systems used in offshore structures are regulated by several standards, among which the ISO 12944:1-8 [15] and the NORSOK M-501 [16] can be found. The selection of the protective system deeply depends on the location of the component. According to ISO 12944-2 [15] and ISO 9223 [17], there are five corrosivity categories; from the C1 corresponding to a non-corrosive atmosphere, to industrial and marine corrosive categories (C5-I and C5-M). There are also IM1 and IM3 categories, which describe the water and soil corrosivity, respectively. The first edition of the ISO 12944-9 [18] was published in January 2018, and adds new categories for offshore-related atmospheres, including: CX-offshore (atmospheric), Im4 (immersion), and CX-Offshore/Im4 for the splash and tidal exposure zones. ISO 12944 also classifies the durability, or the time for the first major maintenance of the protective coatings as:

- **Low (L):** 2 to 5 years
- **Medium (M):** 5 to 15 years
- **High (H):** 15 to 25 years
- **Very High (VH):** >25 years

The categories required for a protective system in an offshore installation are C5-M and Im2, for non-submerged and submerged components, respectively. In order to obtain these protection grades, the ISO 12944 standard recommends employing multilayer coatings with thicknesses between 320–500 µm for a C5-M category in the atmospheric zone, and between 480–1000 µm for the splash zone and submerged components (Im2). According to the standard, these specifications should be enough to ensure a durability of 15 years for a coated component [15]. On the other hand, the NORSOK M-504 standard [16] does not specify a coating thickness range, but it does specify a minimum thickness. Therefore, a coating requiring a C5-M category should be at least 280 µm, whereas submerged components should have a minimum of 350 µm for an Im2 category [16].

The selection of protective coatings for the marine environments strongly depends on the exposure zone and must provide the system with specific properties to assure infrastructures’ durability. The typical coating systems that are employed in offshore applications [19] with the desired properties depending on the exposure zone are compiled in Table 1.

In this work, three coatings used in offshore component protection have been evaluated. The selected coatings are commercial coatings that are currently used in the protection of steel submerged components in mooring line systems. In a previous work, the corrosion and tribocorrosion (wear+corrosion) behavior of the three coatings was evaluated [20]. Furthermore, the characterization of the coatings—in terms of thickness, hardness, porosity, and microscopic analysis of the morphologies—was included in the first part of this work [20]. In the second part of the study, compiled within this document, the corrosion protection effectiveness of the coatings was assessed according to the ISO 12944 and NORSOK M-501 standards by performing weathering aging tests on different cabinets. This work was carried out before the ISO 12944-9 was published, so the conditions and exposure times employed were those in the ISO 12944-6, for the C5-M/Im2 (high) category. On the other hand, a combined aging test was performed in this work according to the ISO 20340 and NORSOK M-501 standards. However, after the publication of the ISO 12944-9, where this test procedure is recommended, ISO 20340 was withdrawn. Therefore, there are no more mentions of the ISO 20340 standard throughout the document, and these tests are referred to the new ISO 12944-9 standard.

## 2. Experimental Procedure

### 2.1. Materials and Sample Preparation

In the present work, the effectiveness of three commercial coatings for offshore structural steel protection has been evaluated. The coatings studied in this work were the following:

- Thermally sprayed carbide (WC–CrCo) with an organic sealant (**C1**)
- Arc thermally sprayed aluminum (TSA) with an organic topcoat (**C2**)
- Epoxy-based organic coating reinforced with ceramic particles (**C3**)

The coatings were applied by the client in the frame of an industrial project, and the information on the coatings composition and preparation is confidential. The three coatings are currently employed in the protection of offshore mooring line components, which are manufactured from high-strength low-alloy (HSLA) steels [7,8,21]. These steels are classified into different grades depending on their ultimate strength: R3, R3S, R4, S4S… [22].

The three coatings evaluated in this work were applied on R4 steel grade panels of 100 × 150 mm^2^, which were mechanized to obtain samples of 100 × 75 mm^2^. The uncoated backside and edges of the panels were isolated with an insulating wax to avoid corrosion of the steel substrate to affect the results. The exposed area of these samples was of 71 cm^2^.

The mean thickness and porosity values measured for the three coating systems in the previous work is presented in Table 2 [20]:

### 2.2. Corrosion Aging Tests

The ISO 12944 and the NORSOK M-501 standards propose several accelerated aging tests such as immersion (ISO 2812-2 [23]), water condensation (ISO 6270 [24]), or salt fog exposure (ISO 9227 [25]); the minimum time for the protective system to be effective depending on the corrosivity category and durability. The NORSOK M-501 and the ISO 12944-9 also propose an accelerated test combining UV irradiation (ISO 16474-3 [26]), salt spray (ISO 9227 [25]), and low-temperature exposure in different chambers, to reproduce the weathering aging conditions of the marine atmosphere. The exposure times that the coatings must fulfill without losing their protective effectiveness to achieve the C5-M and Im2 categories, according to the ISO 12944 standard, are listed in Table 3 for the different weathering aging tests.

In all the tests, visual inspections were performed periodically in order to detect the presence of any defect such as blisters, cracks, delamination of the coating, or corrosion of the substrate. All the samples were kept in the chambers for the maximum duration to achieve the C5-M high or Im2 categories, unless premature failure of the coating due to the appearance of defects was detected (ISO 4628 1-5 [27]).

#### 2.2.1. Immersion Tests (ISO 2812-2)

The resistance of the coatings to immersion was analyzed according to the ISO 2812-2 standard [23] (Table 4). The test was performed in a Julabo ED thermostatic bath (Seelbach, Germany). The temperature of the bath was fixed at 40 °C, and the circulation and aeration system of the water was activated during the whole test duration. The total duration of the test was of 3000 h (Im2-High category). In order to ensure the repeatability of results, three replicates of each coating were tested.

#### 2.2.2. Water Condensation Tests (ISO 6270)

A water condensation test was performed according to the ISO 6270-2 standard [24] to analyze the resistance of the coatings to humidity under water condensation conditions. The standard describes three water atmospheres:

- Condensation atmosphere with constant humidity (CH)
- Condensation atmosphere with alternating humidity and air temperature (AHT)
- Condensation atmosphere with alternating air temperature (AT)

The conditions of the three tests are summarized in Table 4. The test cycles were repeated until a total duration of 720 h was completed. The test was performed in a Kesternich HK300-800 S/M humidostatic chamber (Bielefeld, Germany). Three replicates of each coating were tested at each test atmosphere to ensure the repeatability of results.

#### 2.2.3. Salt Spray Tests (ISO 9227)

Corrosion aging tests in a neutral salt spray chamber were performed under the conditions specified in the ISO 9227 standard [25] (Table 4) in an ASCOTT 2000S chamber (Staffordshire, Great Britain). Six test samples were prepared for each coating. In three of these samples, an X-shaped incision that reached the steel substrate was made (ISO 17872 [28]). No incision was made in the C1 coating due to the high hardness of the tungsten carbide, and the six samples were introduced into the chamber without the scribe. According to the EN ISO 12944-6 standard [15], the corrosion from the scratch in the samples with incisions shall not exceed 1 mm, calculated as:

(1) $$M=\frac{C-W}{2}$$

where W is the original width of the scratch, and C is the maximum width of corrosion across the scratch in millimeters. In the scribed samples, adhesion tests were performed at the end of the exposure, following the ASTM D3359 standard [29].

#### 2.2.4. Combined Aging Test (NORSOK M-501, ISO 12944-9)

The coatings were aged by alternating the exposure to different climatic environments, in accordance with the procedure described in the NORSOK M-501 and ISO 12944-9 standards. The test consisted of combining the exposure to UV radiation and condensation (ISO 16474-3, A method [26]), with the exposure to salt spray (ISO 9227 [25]) and low temperature. The test conditions and duration of each atmosphere is presented in Table 4. The UV/condensation period was performed in an Atlas UV Test weathering device (Illinois, USA). The UV lamps employed in the test were type II UV lamps (UVA-340), of 340 nm (ISO 16474-3). The salt spray and low-temperature periods were carried out in an ASCOTT 2000S chamber and in a WEISS C340/70 climatic chamber (Reiskirchen, Germany), respectively. The UV/condensation period was started with the UV radiation and finished with condensation. The coatings were rinsed with deionized water between the salt spray and the low-temperature periods. The temperature of −20 ± 2 ºC of the low-temperature period was reached in less than 30 min from the moment at which the samples were introduced into the chamber. The cycle was repeated four times, for a total duration of 720 h. The state of the coatings was evaluated three times per cycle, after the coated samples were removed from each chamber.

## 3. Results

The results of the weathering corrosion aging tests for the three coatings in the different climatic chambers, and their categorization in accordance with the ISO 12944 and NORSOK M-501 standards are briefly recapitulated within this section.

### 3.1. Immersion Tests (ISO 2812-2)

**Figure 2:**
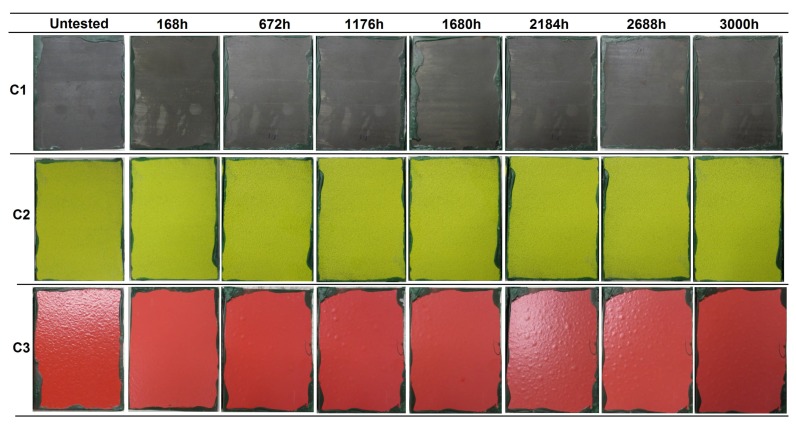
Visual appearance of the R4-coated samples at different evaluations during the immersion test in accordance with the ISO 2812 standard.

Immersion tests were performed to evaluate the resistance of the coatings to water immersion. The surface state of the coatings at different evaluation times is presented in Figure 2, in which one of the three samples of each coating system is presented as representative.

**Figure 3:**
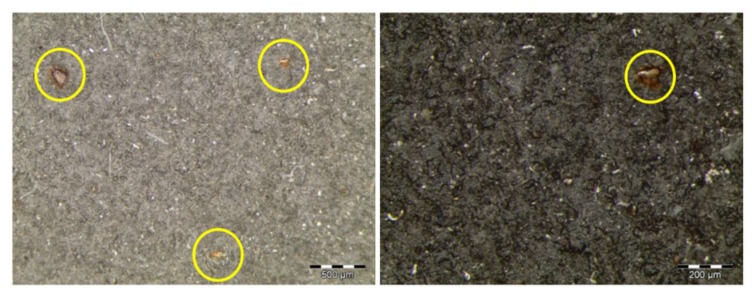
Optical microscopies of the C1 coating surface showing isolated ferric corrosion pits.

In the first evaluation after 168 h of immersion, C1 coating samples presented some decolorized zones in the surface. This could be a consequence of the partial removal of the organic sealant of few µm (Table 2) [20] applied on top of the sprayed carbide. After 336 h, two small ferritic pits were detected in one of the samples. The discoloration of the samples was more evident with increasing exposure time. In the evaluation performed at 1344 h, ferric corrosion was observed in the edges of the samples, close to the insulating wax, which was formed due to a cavitation effect, and by the possible penetration of water under the wax leading to the corrosion of the underneath steel. This corrosion slightly spread with time, but no corrosion sign was detected in the middle zone of the surface. At the end of the test (3000 h), the tone of the tested samples was a more brownish color, and several decolorized and brighter zones were distinguished as a consequence of the sealant loss. The corrosion present in the edges was not considered as a coating failure, since it was observed to be caused by a non-adequate insulating property of the organic wax. The microscopic analysis of the surface revealed some isolated pits along the surface, in a density lower than 1% of the total exposed surface (Figure 3).

In the case of the C2 coating, the presence of ferric corrosion pits was detected close to the edges in the first evaluation at 168 h. These pits corresponded to the oxidation undergone by some metallic particles that were embedded in the surface of the coatings during machining processes, i.e., contaminants. After 672 h, the apparition of white salts resulting from the oxidation of the aluminum layer beneath the organic topcoat was observed. The state of the coating remained unaltered in the following evaluations at longer immersion times. The final surface state of the coating was similar to that of the untested sample, in terms of color and brightness, and no presence of ferric corrosion coming from the steel substrate was detected.

**Figure 4:**
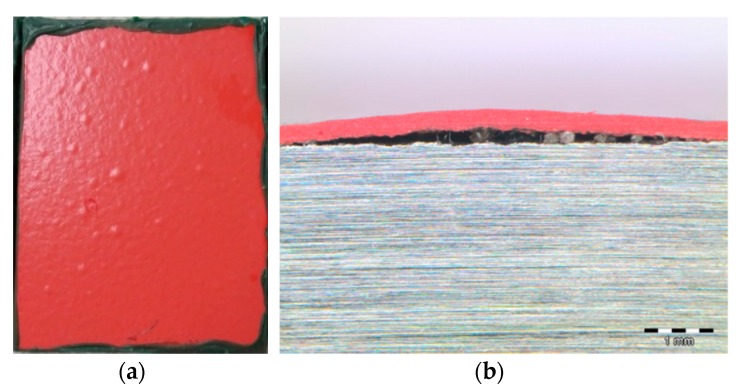
Surface state of sample No.2 of the C3 coating showing blisters after 504 h of immersion (**a**) and micrograph of the ferric corrosion products formed under the blister (**b**).

The samples of C3 coating presented a loss of brightness from the first evaluation at 168 h of exposure, showing a considerably paler tone. After 504 h, blisters appeared in one of the three samples (Figure 4), which was removed from the chamber thereafter. The microscopic analysis of the transversal surface of one of the blisters showed the presence of ferric corrosion products, revealing the underneath corrosion of the substrate by electrolyte penetration through the coating. In the next evaluation, at 672 h, some blisters were detected in the other two samples. The samples were kept in the chamber until 3000 h of immersion were reached, and the blisters were observed to increase in both size and number with higher exposure time. At the end of the test, the surface of the two samples remaining on the chamber was entirely covered with blisters (Figure 2). In accordance with the UNE EN-ISO 4628-2 standard [27], the blistering grade of the three samples was the following:

- C3-sample No.1: 4 (S4)
- C3-sample No.2: 2 (S4) (removed from the chamber after 504 h of immersion)
- C3-sample No.2: 4 (S5)

The C1 and C2 coatings passed 3000 h of immersion, achieving the Im2 (high) category (ISO 12944-6), whereas the C3 coating did not pass the immersion test, not even reaching 2000 h of immersion required for an Im1 (medium) category (see the compilation of results in Table 8 in Section 4).

### 3.2. Water Condensation Tests (ISO 6270)

**Figure 5:**
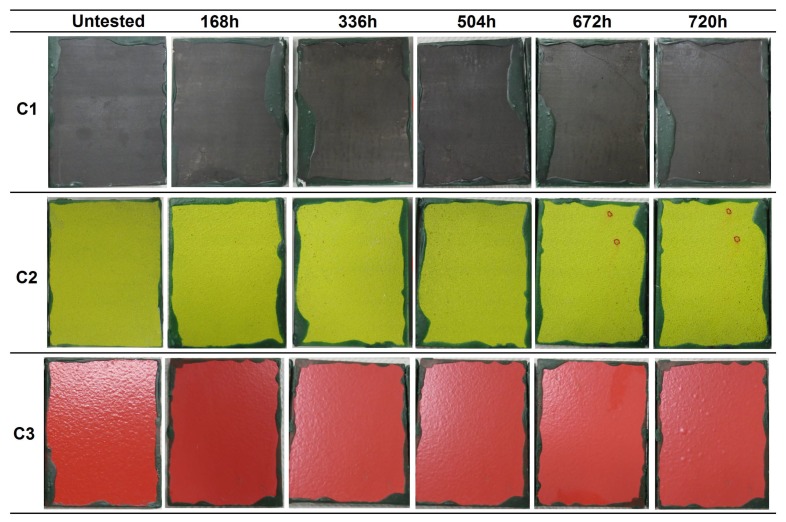
Visual appearance of the coatings’ surfaces at different evaluations during the condensation tests at constant humidity (CH) according to the ISO 6270-2 standard.

**Figure 6:**
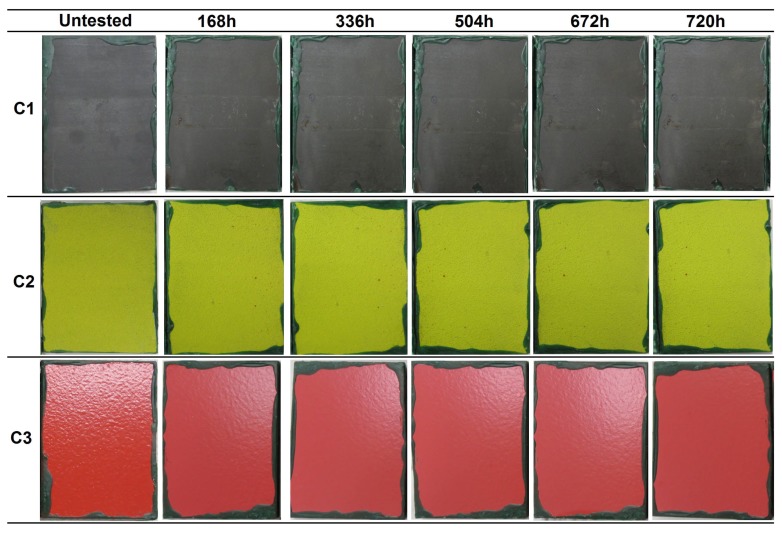
Visual appearance of the coatings’ surfaces at different evaluations during the condensation tests alternating humidity and temperature (AHT) according to the ISO 6270-2 standard.

**Figure 7:**
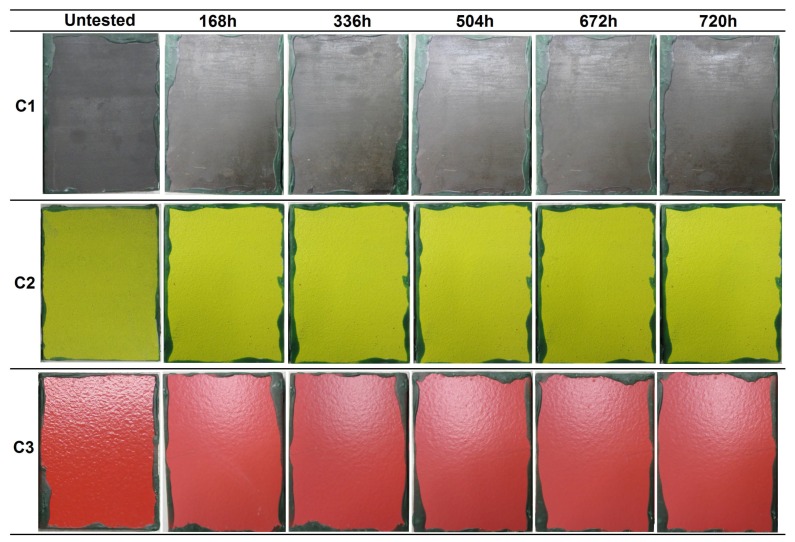
Visual appearance of the coatings’ surfaces at different evaluations during the condensation tests with alternating temperature (AT) according to the ISO 6270-2 standard.

The resistance of the coatings to humidity under water condensation conditions was evaluated under three atmospheres, i.e., constant humidity (CH), alternating temperature and humidity (AHT), and alternating temperature (AT). The evolution of the coatings surface at different evaluation times is presented in Figure 5, Figure 6 and Figure 7 for the CH, AHT, and AT tests, respectively. Table 5 summarizes the results obtained after the condensation tests, expressed as the surface percentage covered by ferric corrosion (FC), white salts (WS), and blistering grade.

In the three exposure atmospheres, the C1 coating presented some decolorized zones in the surface after several hours in the cabinet, which was a consequence of the partial elimination of the sealant applied on top of the sprayed carbide. This was observed after 110 in the CH test, and after 278 h in the AHT and AT tests. Furthermore, ferric corrosion was observed to appear in all the samples close to edges. As previously explained for the samples of the immersion tests, this corrosion phenomenon arose from the cavitation effects in the edges of the insulating wax, and was not considered as a coating failure. In the three test atmospheres, the color of the coating changed with increasing exposure, which was a consequence of the sealant degradation, acquiring a more reddish tone (Figure 5, Figure 6 and Figure 7). In the AHT test, some characteristic red/brown-colored corrosion products were observed in the center of one of the samples, but the surface coverage by these corrosion products did not exceed 1%, so the coating was considered to pass the three tests successfully, with a C5-M (high) category (ISO 12944-6).

In the case of the C2 coating, all the samples presented some red spots on the surface, corresponding to ferric oxides. However, these oxides did not come from substrate corrosion, but rather from the corrosion of metallic particles that were embedded in the coating surface during the sample machining process. No significant changes were observed in the coating during the exposure to any of the three condensation atmospheres in terms of ferric corrosion, blistering, cracking, or color change. In the CH test, the presence of white salt deposits arising from the oxidation of the aluminum layer was detected after 614 h. In the case of the AHT tests, the three coated samples were intact after 720 h of exposure, even without the presence of white salts. Finally, in the AT test, white salt deposits appeared after 336 h in one of the three samples. Therefore, considering the minor changes undergone at the three testing atmospheres, the C2 coating passed the three condensation tests, achieving a C5-M (high) category (ISO 12944-6).

The samples of the C3 coating showed color and brightness loss after several hours of exposure, but no presence of ferric corrosion, cracks, or other defects was detected during the evaluations. The only defects appeared in the CH test samples, which presented a significant part of the surface covered by blisters. However, in the AHT and AT tests, no blister appeared in any of the coated samples after 720 h in the chamber. According to the UNE EN-ISO 4628-2 standard [27], the blistering grade of the three samples of the CH test was the following:

- C3-sample No.1: 2 (S3)
- C3-sample No.2: 2 (S4)
- C3-sample No.2: 2 (S4)

Consequently, the C3 coating was classified within the C5-M (high) category for the AHT and AT tests, and with a C4 (high)/C5-M (medium) category for the CH test, corresponding to an optimal surface condition of the coating for 480 h of exposure to constant humidity (ISO 12944-6) (see Table 8 in Section 4 for the compilation of results).

### 3.3. Salt Spray Tests (ISO 9227)

**Figure 8:**
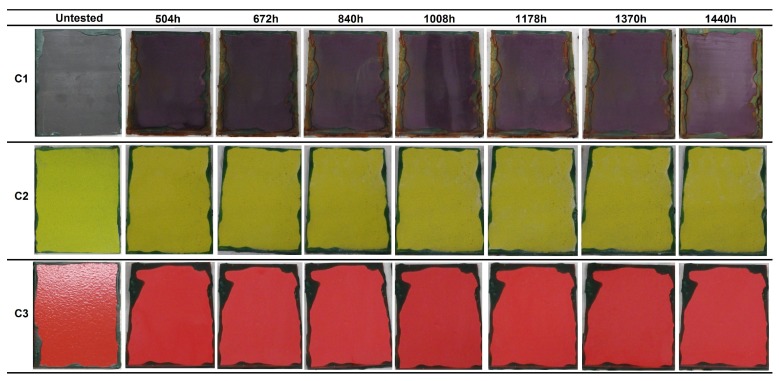
Visual appearance of the coatings’ surfaces without incision at different evaluations during 1440 h of exposure to salt spray, according to the ISO 9227 standard.

**Figure 9:**
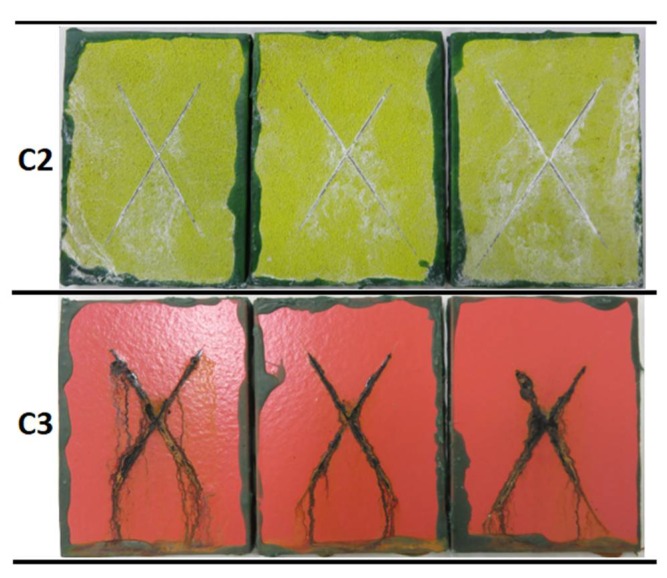
Visual appearance of the coatings surface without incision at different evaluations during 1440 h of exposure to salt spray, according to the ISO 9227 standard.

The coatings were exposed to neutral salt spray conditions to evaluate the resistance to a saline corrosive environment. The evolution of the surface state of the three coatings without incision during the exposure to salt spray atmosphere at different evaluation times is depicted in Figure 8. The final appearance of the scribed samples after 1440 h in the chamber is shown in Figure 9 for the C2 and C3 coatings. Table 6 summarizes the degradation observed in the coatings for the different evaluations, in terms of the surface percentage covered by ferric corrosion or white salts in the samples without incision, and the progression of the ferric corrosion on the scribed coatings.

**Figure 10:**
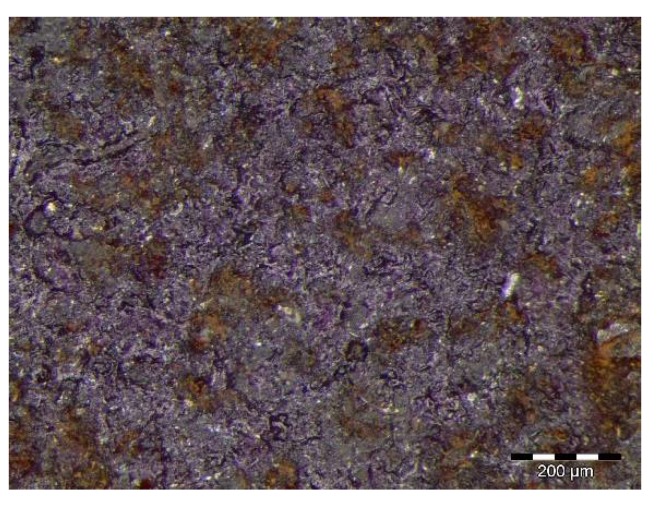
Surface estate of the C1 coating after 1440 h of exposure to neutral salt spray showing a high density of ferric corrosion pits along the whole exposed surface.

The C1 coating remained unaltered for the first 504 h of exposure, after which corrosion was observed to spread in all the samples. After 1440 h in the chamber, four of the six samples presented with 25–30% of the surface covered by ferric corrosion. The color of the coating changed significantly with exposure time, acquiring a purple tonality. The surface analysis by means of a magnifying glass revealed the presence of ferric pits that were homogeneously distributed along the whole exposed surface (Figure 10). The oxidation grade of the coating was Ri4 (S2), in accordance with the designation of the UNE-EN ISO 4628-3 standard [27] for a rusted area of around the 8% of the exposed surface.

In the case of the C2 coating, the presence of white salts was detected after 672 h in the chamber. These salts were observed in the form of small deposits, which increased in size with exposure time. The surface percentage covered by white salts was 5% after 1178 h and increased, reaching the 5–10% at the end of the test. The samples that had a C2 coating with an incision did not present ferric corrosion in any evaluation. White salts were observed from the first evaluation at 504 h. The adhesion test results obtained for the scribed samples at the end of the aging test was of 5A grade for two of the three samples, corresponding to the higher grade of the scale, with no detachment of the coating. The third sample was classified as 4A, since a small trace of the coating was removed with the adhesive tape.

The C3 coating remained unaltered for 840 h, at which point the presence of one blister was detected in two of the three samples without incision, but the apparition of new blisters or other defects was not observed in the following evaluations. The third sample remained unaffected until the end of the test. The samples with incision showed the progression of ferric corrosion from the first evaluation at 504 h. Ferric corrosion spread with exposure time, exceeding 2 mm in two of the three samples after 1370 h. Furthermore, a blister was detected in one of these samples. The adhesion results were 5A grade for two of the samples, and the third was 4A grade.

Considering the high amount of ferric corrosion in the C1 coating, and the presence of a blister in the C3 coating and the corrosion exceeding 1 mm from the scratch on the scribed samples, the failure of these two coatings was estimated at 504 h and 672 h, respectively. Therefore, they were classified as belonging to the C4 (medium)/C5-M (low) categories (ISO 12944-6). However, the C2 coating overcame the 1440 h of exposure to salt spray, showing no ferric corrosion coming from the steel substrates, and proving an effective protection in such an aggressive atmosphere. Thus, the C2 coating was classified as belonging to the C5-M (high) category (ISO 12944-6) (see Table 8 in Section 4 for the compilation of results).

### 3.4. Combined Aging Test (NORSOK M-501, ISO 12944-9)

**Figure 11:**
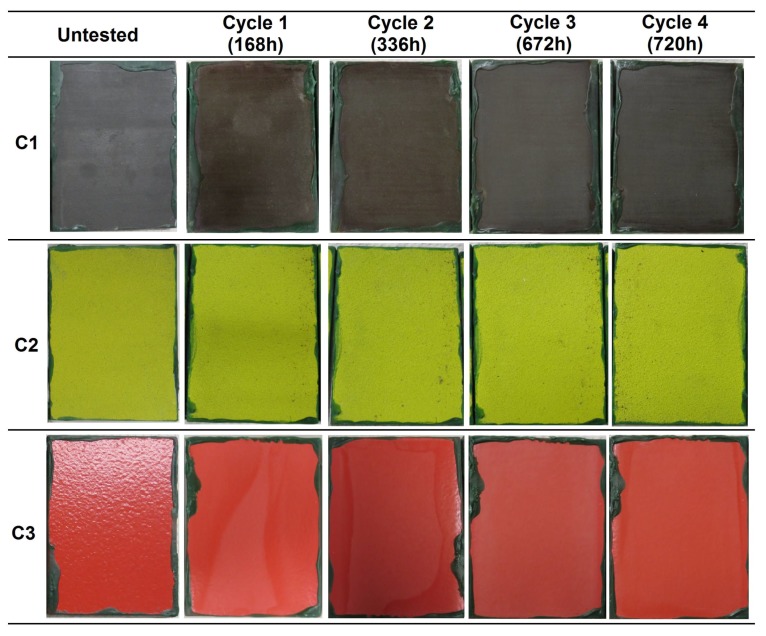
Visual appearance of the coatings’ surfaces after 720 h of exposure to combined aging according to the ISO 12944-9 and NORSOK M-501 standards.

In the combined aging test, the coatings were exposed to different atmospheres, so as to reproduce the climatic conditions of the marine environment. The samples were subjected to UV radiation, water condensation, salt spray, and low temperature. The evolution of the surface state of the three coatings at different exposition times is depicted in Figure 11. The degradation degree in terms of the ferric corrosion coverage of the samples and blistering are summarized in Table 7.

**Figure 12:**
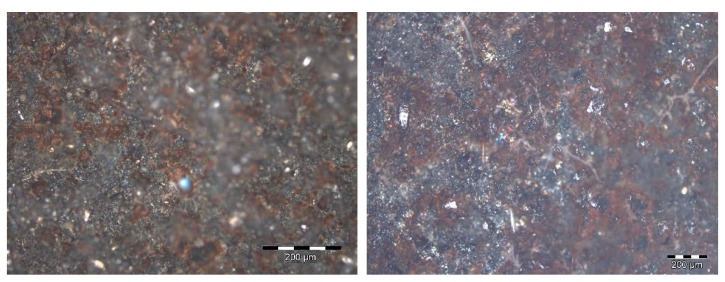
Micrographs at different magnifications of the surface estate of the C1 coating after 720 h of exposure to combined aging presenting ferric pits along the whole exposed surface.

Ferric corrosion was observed in the samples coated with C1 from the end of the exposure to salt spray in the first test cycle, which covered 1% of the total exposed surface in the three specimens. This corrosion appeared close to the edges of the samples, as a consequence of the cavitation effects of the insulating wax that was employed. The three samples lost their initial brightness, which was a consequence of the degradation and elimination of the organic sealant. Ferric corrosion increased during the second test cycle, and the entire surface of the samples acquired a more reddish tone. This tonality change undergone by the coating was found to be the consequence of the presence of ferric corrosion pits that were evenly distributed along the whole exposed surface of the samples (Figure 12), which exceeded 10% of the exposed area in two of the three samples.

In the case of the C2 coating, oxides coming from metallic impurities were observed, which was similar to the samples employed in the previous tests. Some white salts arising from aluminum oxidation were detected in one of the samples during the second cycle, but no further changes were observed in any of the samples during the remaining tests cycles. Neither significant color and appearance alterations, nor the apparition of ferric corrosion or other defects were observed at the end of the test.

The C3 coating showed discoloration and a significant loss of brightness from the first exposure to UV radiation, especially at the center of the samples. The color change to a paler tone was more noticeable with increasing exposure to weathering conditions, but no other defects such as blisters or ferric corrosion were observed in any sample.

Considering the significant number of ferric corrosion pits present on the surface of the samples, the C1 coating did not pass the combined aging test. On the other hand, the C2 and C3 coatings passed the test, showing just some discoloration and brightness loss in the case of the C3 coating, which does not compromise the protective performance of the coating (Table 7).

## 4. Discussion

After performing the different weathering tests, with the durations for the C5-M (H) and Im2 (H) categories specified in the ISO 12944-6 standard corresponding to high corrosivity atmospheres, the results obtained for the three coating systems evaluated in this work is summarized in Table 8.

The thermal sprayed carbide layer with a thin organic sealant of few nanometers (C1) was found to present ferric corrosion pits in some of the testing atmospheres. Coherently with the results obtained for the C1 coating in a previous work, in which the electrochemical corrosion response of the coating was evaluated in synthetic seawater [20], the organic sealant was found to deteriorate, losing its protective ability in a relatively short period of exposure to the corrosive environment. In this work, the loss of the sealant was observed in most of the weathering atmospheres, after which the brightness of the samples and/or their color changed. After the sealant degradation took place, the underneath sprayed layer was exposed to the corrosive atmosphere. Sprayed coatings usually present a morphology containing pores and other defects, which is a consequence of the solidification of the sprayed material between successive splats and the thermal stresses that might generate during the spraying process [30,31,32]. The morphological characterization of the carbide sprayed layer composing the C1 coating (which was evaluated in the previous work [20]) revealed a structure with pores and cracks. These defects constitute ion-conducting paths across the coating, which allow the migration of water and aggressive species to the coating/substrate interface, eventually leading to the corrosion of the steel underneath. In the weathering aging tests, this was observed as the apparition of ferric corrosion pits in some of the test samples. In the tests in which the isolated corrosion pits were detected, the coatings were considered to provide enough protection to the steel substrate overcoming the test conditions, as in the case of the immersion tests and the condensation tests at the three atmospheres (CH, AHT, and AT). In the cases that presented a higher density of corrosion pits, with a considerable coverage of the exposed surface, the coating was not considered to reach the maximum time specified for the C5-M (H)/Im2 (H) category, e.g., the salt spray and the combined aging tests.

In the case of the arc thermally sprayed aluminum with an organic topcoat (C2), this was the only protective system that overcame all the weathering tests successfully. In this coating system, the thickness of the organic topcoat of several nanometers acted as an adequate barrier against the aggressive environments evaluated. In some of the tests, e.g., the immersion tests, the condensation tests (CH and AT), and the salt spray tests, white deposits corresponding to aluminum oxides were detected. In these tests, which constituted high humidity atmospheres during the whole exposure period, water penetrated through the conductive paths generated in the organic coating. Once the humidity reached the sprayed layer, the aluminum reacted to form aluminum oxides or hydroxides in the form of white salts. These results are in coherence with those observed in previous works in which the thermally sprayed aluminum layer with [20] and without [21] the organic topcoat was immersed in synthetic seawater for several weeks to perform the electrochemical corrosion tests. In this works, aluminum hydroxides were identified to form on the surface of the aluminum layer once this layer was exposed to the corrosive atmosphere. Nevertheless, the corrosion of the aluminum was not a sign of the degradation of the coating, but rather constituted an improvement as a barrier against aggressive species, by blocking both the pores and defects present in the sprayed layer as well as the conductive paths in the organic topcoat [20,21]. Therefore, considering the appearance of white salts in the coated panels tested in this work, but the absence of ferric corrosion coming from the substrate degradation, the two-layered coating provided the steel substrate with an adequate protection against corrosion in the different weathering atmospheres evaluated in this work. Furthermore, the current use of aluminum coatings in submerged components is related to the sacrificial corrosion undergone by the metal in contact with steel due to the galvanic coupling generated in seawater. Consequently, in view that the C2 coating resisted the highest test duration in all the weathering atmospheres, this coating system can be expected to last for high periods of exposure to the marine environment thanks to the sacrificial protection provided by the aluminum layer.

Finally, the organic coating reinforced with ceramic platelets (C3) presented blistering in the tests comprising the highest humidity atmosphere during the whole test duration, i.e., the immersion, condensation (CH), and salt fog tests. The formation of blisters is closely related to the delamination of organic coatings on the coating/substrate interface as a consequence of a low adhesion of the coating allowing the penetration of water. In the electrochemical corrosion tests performed in the previous work for this coating [20], the corrosion resistance of the coating decreased with higher immersion times in synthetic seawater, which was a consequence of the penetration of seawater into the coating/substrate interface. Organic coatings have been so far the best protective solution against the corrosion of structural steel in marine atmospheres, due to their organic nature with low electrical conductivity, which constitutes an effective barrier to corrosive species [33,34]. However, the low adhesion of the coating can lead to the quick penetration of water and delamination of the coating due to the underneath corrosion of the substrate. In this work, the optical evaluation performed in the transversal surface of one of the blisters revealed the presence of ferric corrosion products. Therefore, the unexpected poor results obtained for the organic coating in some of the weathering tests might be related to a low adherence of the coating due to the inadequate surface cleanliness of the metallic substrate prior to the coating application.

## 5. Conclusions

In the present work, the effectiveness of three coatings for the corrosion protection of offshore structural steel has been evaluated, according to the ISO 12944 and NORSOK M-501 standards. The corrosivity category of the coatings was determined for each aging test condition (ISO 12944). The conclusions of this work were the following:

- From the immersion tests (ISO 2812-2), the only coating that did not reach 4000 h of immersion was the C3 coating. Blisters were detected to appear after 504 h in one of the samples, and after 672 h in the other two samples. The C1 and C2 coatings were classified as IM2 (high), overcoming the 4000 h of exposure without the appearance of any defect.
- Regarding the water condensation tests (ISO 6270), the C1 and C2 coatings obtained the C5-M (high) category in the three water atmospheres. However, C3 was classified as C5-M (high) in the AHT and AT atmospheres, and as C4 (high)/C5-M (medium) for the CH test due to the apparition of blisters after 614 h of exposure.
- In the case of the salt spray tests (ISO 9227), C2 was the only coating reaching the C5-M (high) category. The C1 and C3 coatings were classified as C4 (medium)/C5-M (low), after the detection of ferric corrosion (480 h) in the C1 coating and blisters (972 h) in the C3 coating. The adherence that was measured for the C2 and C3 coatings in the samples with incision was 4A/5A, according to the ASTM D3359 standard. The adherence of the C1 coating was not measured, since the incision could not be made in the hard carbide layer.
- With respect to the combined aging tests (NORSOK M-501, ISO 12944), the C1 coating did not pass the test, since the surface of the samples was covered by a considerable amount of ferric corrosion at the end of the last test cycle. On the other hand, the C2 and C3 coatings passed the test, showing just some discoloration and loss of brightness, maintaining the protective properties.
- Finally, the only coating that reached the higher category and overtook all the test atmospheres was the C2 coating. The combination of both the aluminum layer and the organic topcoat providing a two-layered protection by means of a sacrificial aluminum anode and the high corrosion resistance of the organic layer.

## Figures and Tables

**Table 1:** Typical coating systems employed in offshore applications in the different exposure zones [19], with the desired properties and corrosivity category required (ISO 12944 [15]).

Exposure Zone	Category (ISO 12944)	Coating System		Desirable Coating Properties
**Atmospheric**	C5-M	Zinc-rich epoxy primer	(60–100 µm)	Corrosion-resistant, erosion-resistant, anti-icing, UV-resistant
		Epoxy intermediate layer	(100–120 µm)	
		Polyurethane top coat	(50–80 µm)	
**Splash and tidal**	C5-M and Im2	Two or three epoxy-based coats	(>1000 µm in total)	Combination of atmospheric and submerged coatings´ properties
		Polyurethane top-coat	(50–80 µm)	
**Submerged**	Im2	Two or three epoxy-based coats	(>450 µm in total)	Corrosion-resistant, antifouling, wear-resistant

**Table 2:** Mean thickness and porosity values measured for the three coatings [20]. C1: a thermally sprayed carbide coating with an organic sealant; C2: a thermally sprayed aluminum (TSA) with an organic topcoat; C3: an epoxydic organic coating reinforced with ceramic platelets.

Coating	Thickness (µm)		Porosity (%)
	Organic Sealant	Sprayed Metal	
**C1**	9 ± 3	191 ± 14	1.9 ± 0.4
**C2**	239 ± 8	284 ± 14	4.7 ± 0.3
**C3**	430 ± 15 *		-

* The thickness of the C3 coating corresponds to the total thickness of the epoxydic layer with ceramic platelets.

**Table 3:** Minimum exposure time for the coatings to achieve the C5-M and Im2 categories according to the ISO 12944 standard [15].

Category	Durability Ranges	ISO 2812-2 [23] (Immersion in Water)	ISO 6270-2 [24] (Water Condensation)	ISO 9227 [25] (Salt Spray Test)
**C5-M**	Low	-	240 h	480 h
	Medium	-	480 h	720 h
	High	-	720 h	1440 h
**Im2**	Low	-	-	-
	Medium	2000 h	-	720 h
	High	3000 h	-	1440 h

**Table 4:** Summary of the weathering corrosion aging test conditions in accordance with the different standards, and test duration for the C5-M and Im2 categories [15].

Standard	Test Type		Cycle Duration			Conditions in the Cabinet		Total Duration of the Test
			Test Periods		Total	Temperature	Relative Humidity	
**ISO 2812** [23]	Immersion test		-			(40 ± 3) °C	-	3000 h
**ISO 6270** [24]	Constant humidity condensation atmosphere (CH)		From warm-up to end of exposure			(40 ± 3) °C	100%	720 h
	Alternating condensation atmosphere	Alternating humidity and air temperature (AHT)	8 h including warm-up		24 h	(40 ± 3) °C	100%	720 h
			16 h including warm-up			18–28 °C	Ambient	
		Alternating air temperature (AT)	8 h including warm-up		24 h	(40 ± 3) °C	100%	720 h
			16 h including warm-up			18–28 °C	100%	
**ISO 9227** [25]	Salt spray		-			(35 ± 3) °C	-	1440 h
**NORSOK M-501** [16] **ISO 12944-9** [18]	Ultraviolet radiation and condensation (ISO 16474-3 [26])		4 h UV radiation (0.77 W/m^2^)	72h	168 h	(60 ± 3) °C	-	720 h
			4 h condensation			(50 ± 3) °C	100%	
	Salt spray (ISO 9227 [25])		72 h			(35 ± 3) °C	-	
	Low temperature		24 h			(−20 ± 2) °C	-	

**Table 5:** Summary of the oxidation and blistering grade of the coatings after the condensation tests in accordance with the ISO 6270-2 standard [24] regarding the percentage of damaged surface.

Coating		Oxidation Grade				Blistering Grade		
		CH	AHT	AH		CH	AHT	AH
C1	Sample 1	3% (*E)	2% (E)	3% (E)		0%	0%	0%
	Sample 2	3% (E)	2% (E) + 1% (**C)	2% (E)		0%	0%	0%
	Sample 3	3% (E)	1% (E)	-		0%	0%	-
C2	Sample 1	1% (***WS)	0%	0%		0%	0%	0%
	Sample 2	1% (WS)	0%	1% (WS)		0%	0%	0%
	Sample 3	1% (WS)	0%	-		0%	0%	-
C3	Sample 1	0%	0%	0%		20%	0%	0%
	Sample 2	0%	0%	0%		30%	0%	0%
	Sample 3	0%	0%	-		30%	0%	-

* E: ferric corrosion at edges; ** C: ferric corrosion at the center; *** WS: white salts.

**Table 6:** Degradation observed in samples with the three coatings with and without incision at different evaluation times during exposure to a salt spray chamber (ISO 9227 [25]) and the adhesion test results of the samples with incisions (ASTM D3359 [29]).

Coating	Incision	Sample No.	Exposure Time (h)							Adhesion Test (ASTM D3359)
			504	672	840	1008	1178	1370	1440	
**C1**	No	1	~15% FC (*)	25% FC	25% FC	25% FC	25% FC	25% FC	25% FC	-
	No	2	~5% FC	5% FC	5% FC	5% FC	5–10% FC	5–10% FC	5–10% FC	-
	No	3	15-20% FC	25% FC	25% FC	25% FC	25–30% FC	~30% FC	~30% FC	-
	No	4	15% FC	20% FC	~20% FC	~20% FC	~30% FC	~30% FC	~30% FC	-
	No	5	Isolated pits	<5% FC	<5% FC	<5% FC	<5% FC	<5% FC	<5% FC	-
	No	6	10–15% FC	15–20% FC	20–25% FC	20–25% FC	20–25% FC	20–25% FC	20–25% FC	-
**C2**	No	1	Unaltered	Ws (*) + P (*)	Ws + P	Ws + P	<5% Ws	<5% Ws	5-10% Ws	-
	No	2	Unaltered	Unaltered	Unaltered	Ws + P	<5% Ws	<5% Ws	5% Ws	-
	No	3	Unaltered	Ws + P	Ws + P	Ws + P	<5% Ws	<5% Ws	5–10% Ws	-
	Yes	4	Ws + Np (*)	Ws + Np	Ws + Np	Ws + Np	Ws + Np	Ws + Np	Ws + Np	5A
	Yes	5	Ws + Np	Ws + Np	Ws + Np	Ws + Np	Ws + Np	Ws + Np	Ws + Np	4A
	Yes	6	Ws + Np	Ws + Np	Ws + Np	Ws + Np	Ws + Np	Ws + Np	Ws + Np	5A
**C3**	No	1	Unaltered	Unaltered	1 blister	1 blister	1 blister	1 blister	1 blister	-
	No	2	Unaltered	Unaltered	1 blister	1 blister	1 blister	1 blister	1 blister	-
	No	3	Unaltered	Unaltered	Unaltered	Unaltered	Unaltered	Unaltered	Unaltered	-
	Yes	4	0.4-mm PFC (*)	0.6-mm PFC	0.6-mm PFC	0.8-mm PFC	0.8-mm PFC	2-mm PFC	2-mm PFC	4A
	Yes	5	0.5-mm PFC	0.8-mm PFC	0.8-mm PFC	0.8-mm PFC	0.8-mm PFC	1.2-mm PFC	1.2-mm PFC	5A
	Yes	6	0.5-mm PFC	0.9-mm PFC	0.9-mm PFC	1-mm PFC	1-mm PFC	2.6-mm PFC + 1 blister	2.6-mm PFC + 1 blister	5A

(*) FC = Ferric corrosion; Ws = White salts; P = Pits; Np = No progression of ferric corrosion; PFC = progress of ferric corrosion.

**Table 7:** Summary of the oxidation and blistering grade of the coatings after the combined aging test in accordance with the ISO 12944-9 [18] and NORSOK M-501 [16] standards.

Coating		Oxidation Grade	Blistering Grade
**C1**	**Sample 1**	16%	0%
	**Sample 2**	4%	0%
	**Sample 3**	12%	0%
**C2**	**Sample 1**	2% (Inclusions)	0%
	**Sample 2**	2% (Inclusions)	0%
	**Sample 3**	3% (Inclusions)	0%
**C3**	**Sample 1**	0%	0%
	**Sample 2**	0%	0%
	**Sample 3**	0%	0%

**Table 8:** Summary of the corrosivity categories of the ISO 12944-6 [15] standard obtained for the three coatings in all the weathering aging tests performed.

Test/Standard		Coating Category (ISO 12944-6)		
		C1	C2	C3
**Immersion (ISO 2812)**		Im2 (High)	Im2 (High)	No pass (Blistering)
**Condensation** **(ISO 6270)**	**CH**	C5-M (High)	C5-M (High)	C4 (High)/C5-M (Medium)
	**AHT**	C5-M (High)	C5-M (High)	C5-M (High)
	**AT**	C5-M (High)	C5-M (High)	C5-M (High)
**Salt spray (ISO 9227)**		C4 (Medium)/C5-M (Low)	C5-M (High)	C4 (Medium)/C5-M (Low)
**Combined aging** **(ISO 12944-9, NORSOK M-501)**		No passed	Passed	Passed
